# Acute Effects of Kinesio Taping on Functional Performance in Healthy Soccer Players: A Randomized, Controlled Crossover Trial

**DOI:** 10.3390/jfmk8010002

**Published:** 2022-12-20

**Authors:** Giuseppe Annino, Anas Alashram, Cristian Romagnoli, Emanuele Balducci, Marco De Paolis, Vincenzo Manzi, Elvira Padua

**Affiliations:** 1 Department of Systems Medicine, Faculty of Medicine and Surgery, University of Rome “Tor Vergata”, 00133 Rome, Italy; 2 Department of Physiotherapy, Middle East University, Amman 11831, Jordan; 3 Sport Engineering Lab, Department of Industrial Engineering, University of Rome “Tor Vergata”, 00133 Rome, Italy; 4 School of Human Movement Science, Faculty of Medicine and Surgery, University of Rome “Tor Vergata”, 00133 Rome, Italy; 5 Department of Humanities Science, Pegaso Open University, 80143 Naples, Italy; 6 Department of Human Sciences and Promotion of the Quality of Life, San Raffaele Roma Open University, 00133 Rome, Italy

**Keywords:** functional performance, athletic training, sport, rehabilitation, Kinesio tape

## Abstract

This study aimed to investigate the acute effects of the Kinesio tape (KT) application on functional performance in healthy athletes. In this randomized, controlled crossover trial, a total of sixteen healthy soccer players (male = 14; age = 23.28 ± 3.13 years old) were assigned randomly into either KT over quadriceps, KT over hamstring, KT over quadriceps plus hamstring, or no intervention control condition. Four conditions were applied in a crossover design through three consecutive test sessions for each condition with a washout period of 2 days between the trials. Afterwards, all participants performed a 5-min warm-up routine and four sets of 30-s static stretching exercises for the hamstring and gluteal muscles for three consecutive treatment sessions. The running, jump, and flexibility tests were used to assess the functional performance of healthy athletes. A total of sixteen participants completed the study. No significant differences in the jump, flexibility, and running tests among the conditions were reported (*p* > 0.05). These findings suggest that KT application has no acute effects in improving functional performance in healthy athletes. However, further studies with larger sample sizes are needed to verify our results.

## 1. Introduction

The Kinesio taping (KT) technique was created by Dr Kenso Kase in the 1970s [1]. This technique is based on a thin adhesive elastic tape application that can be stretched up to 55–60% of its original length [2,3]. The beneficial effects of KT include physical corrections, space recuperation, movement rectification, fascia relaxation, ligament and tendon support, lymphatic fluid circulation, and proprioception stimulation [4,5,6,7,8,9]. KT application provides constant tension to the skin transmitted to the skeletomuscular fascia [10]. Lumbroso et al. (2014) suggest the increase of cutaneous tension from KT application as a possible reason for increased gastrocnemius and hamstring muscle strength and the increased range of motion of the knee and ankle joints [11].

Sports performance is the result of the interaction of numerous mental and physical factors. One of the main factors in improving and maximizing athletic performance is the development of physical abilities. Functional performance consists of essential components such as jumping, strength, power, endurance, and flexibility [12,13,14]. The impact of taping in sports is still an ongoing discussion in academia. Objectors have downplayed their influence on either musculoskeletal injury [15,16] or spinal pain [17]. Alternatively, those in favour have suggested positive effects of taping on patellofemoral pain syndrome [18,19], shoulder impingement syndrome [20], rotator cuff tendinitis [21], and lower back pain [22]. Nonetheless, some evidence could still indicate the effect of taping on rehabilitation extent.

Apart from the influence of taping on musculoskeletal disorders recovery, evidence has suggested an alternative perspective regarding sports performance enhancement [23,24]. Many studies have shown the positive effects of taping on the horizontal jump, sprint cycling speed, and balance performance [25,26,27]. Despite the benefit of taping on functional performance still being doubted [28], further explanations about the effects of different types of tape on performance have been demanded to develop a comprehensive interpretation.

In terms of KT, many experimental studies have demonstrated an increasing range of motion, power, and flexibility after applying KT to quadriceps muscles in healthy non-athlete individuals [29,30,31]. Recently, a systematic review by Nunes et al. (2020) did not support the use of Kinesio taping applied to the ankle for improvements in functional performance in people with or without ankle injuries [32]. Another systematic review reported a paucity of compelling evidence to support the immediate effects of KT on muscular performance in soccer players, handball players, and runners [33]. Therefore, this study aimed to investigate the acute effects of KT application on functional performance in healthy soccer players.

## 2. Materials and Methods

### 2.1. Participants

A total of sixteen male professional healthy soccer players participated in this study (mean age = 23.28 ± 3.13 years old; height, 178.93 ± 1.79 cm; weight, 73.17 ± 7.84 kg). The body mass and height of the subjects were measured to the nearest 0.5 kg and 0.5 cm, respectively (Seca Beam Balance-Stadiometer, Hamburg, Germany). All participants were informed of the study protocol and provided their informed consent to participate. The inclusion criteria were as follows: no history of lower limb injuries in the last 12 months and having practiced the sport for at least one year before the present experiment. The participants were excluded from this study if they had current musculoskeletal injuries, serious cardiopulmonary diseases, or had experienced physical discomforts, such as seizures and severe dizziness. This study was approved by the Internal Research Board of “Tor Vergata” University of Rome. All procedures were carried out in accordance with the Declaration of Helsinki.

### 2.2. Experimental Design

This is a randomized, controlled crossover trial. The participants were assigned randomly into either Kinesio taping over quadriceps (Q), Kinesio taping over hamstring (H), Kinesio taping over quadriceps plus hamstring (H + Q), or no intervention control condition (no tape). Four conditions were applied in a crossover design through four consecutive test sessions for each condition with a washout period of 2 days between the trials.

Next, all participants in each condition were asked to perform a 5-min warm-up routine (i.e., cycling without resistance) followed by four sets of 30-s static stretching exercises for the hamstring, quadriceps, and gluteal muscles, with a 30-s rest interval between stretch sets. The participants in each condition performed three consecutive treatment sessions.

#### Tape Application Criteria

The facilitation taping application described by Kase et al. (2003) was implemented [3]. The tape was applied from the muscle origin to the insertion (proximal to distal). The tape endpoint was rounded to prevent the edges from peeling off and increase the tape application length.

**Figure 1:**
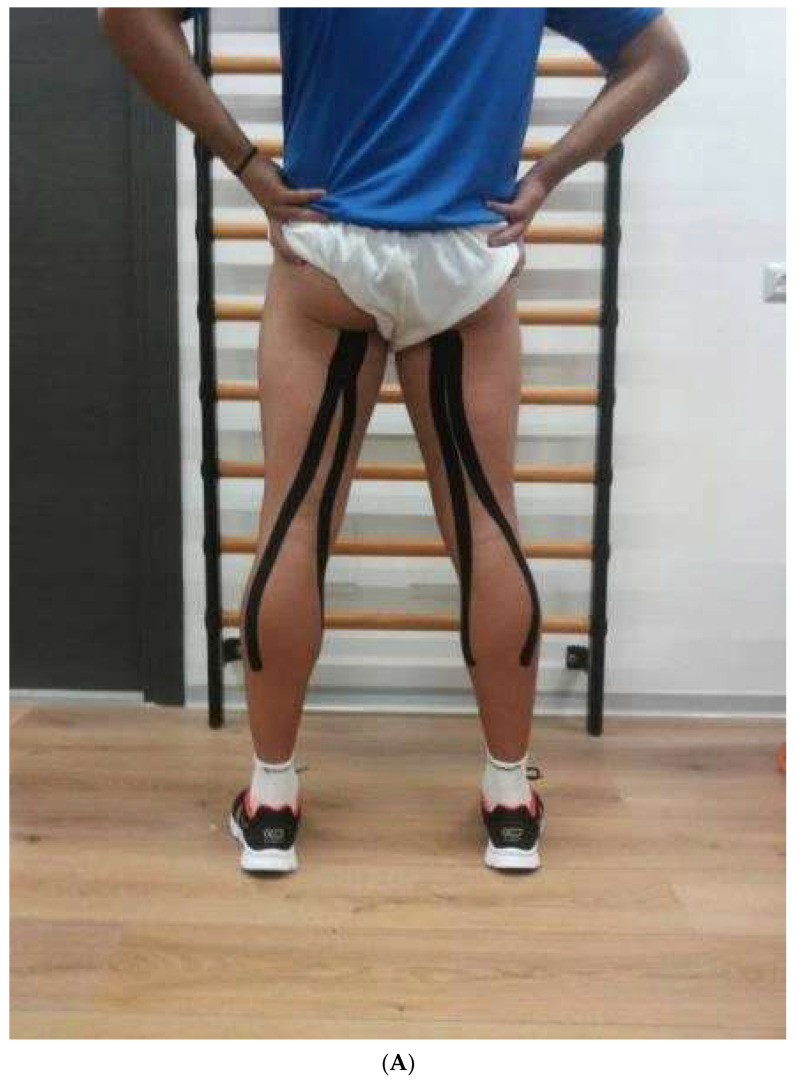

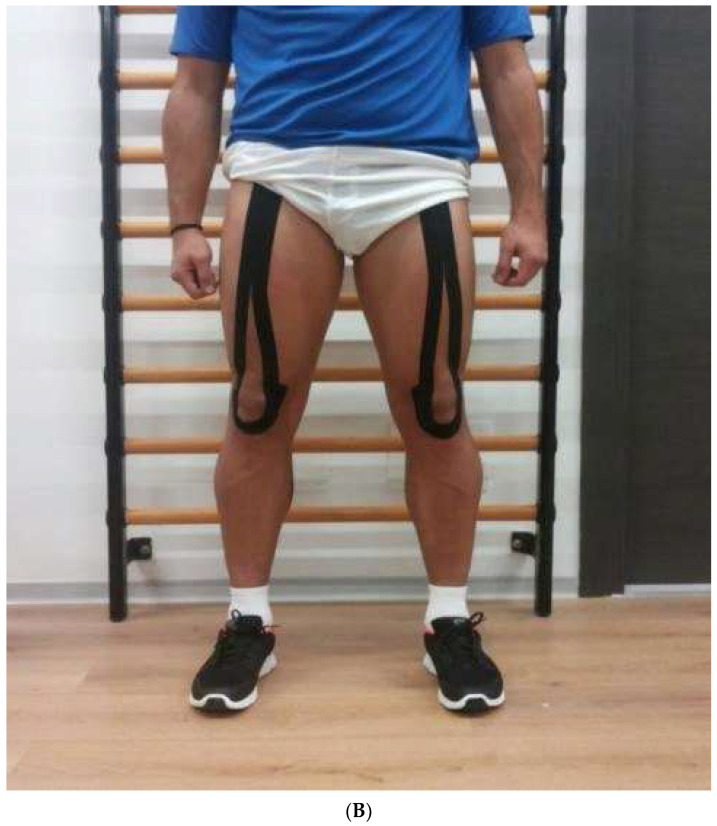
(**A**) Hamstring muscles tape application. (**B**) Quadriceps muscles tape application.

Hamstring muscle tape application was applied from the proximal side of the muscle, passing down to the lateral and medial heads of the gastrocnemius muscle (Figure 1A). On the quadriceps, the tape was applied from the proximal side of the muscle, passing down to the inferior surface of the patella (Figure 1B).

The therapist placed the base of the tape strip 5 cm below the origin of the hamstring and quadriceps muscles without tension. Light to moderate tension (25–50%) was then applied along with the tails. When the tails were 2 to 5 cm from the end, they were placed on the skin without any tension. The tape was applied by a specialist therapist in the same way.

### 2.3. Testing Procedure

One assessor was blinded to the participants who performed the tests after each session. The participants were wearing sweatpants, so the assessor could not know the participants’ groups. The run, jump, and flexibility tests were performed in each condition.

#### 2.3.1. Jump Test

The countermovement jump test (CMJ) was used as a primary outcome measure to evaluate the explosive force. The test was performed using a contact platform, hardware interface, and the application software Chrono Jump^®^ (Reliability: ICC = 0.86; 95% CI (0.54–0.96)) [32]. The participants performed two sets of three countermovement jumps (CMJ), each with hands resting on the hips. The resting time between jumps was 10 s, with 3 min intervals between two sets. The best jump was used for statistical analysis.

#### 2.3.2. Running Test

A running test was performed using the Opto Jump performance analysis system (Reliability: Fly Time, ICC = 0.93; 95% CI (0.890–0.957), Contact Time, ICC = 0.97; 95% CI (0.946–0.979)) [34]. The system consists of two bars: the transmitting and receiving bars (each bar has 96 LEDs; 1.041 cm resolution). The LEDs of the transmitting bar relate to those on receiving the bar. The system detects potential interruptions and calculates the duration of these interruptions that allow measures of flying and contacting times. In the present study, the participants performed four running sessions with 2 min for each, at the speed of 11 km/h. From the analysis results, an average of four parameters were adopted. The results given by the software included the time of contact, time of flight, stride, the width of stride [cm], and rhythm of steps per second [p/s].

#### 2.3.3. Test of Flexibility

Hamstring and lower back flexibility were evaluated using the “sit and reach” test (Reliability: ICC = 0.98; 95% CI (0.970–0.990)) [35]. The participants were sitting with their knees straight and feet flat against the front end of the box test. The participants were asked to lean forward at the hips in a slow, steady movement, keeping the knees straight and sliding the hand up the box ruler as far as they could go, extending as far as they could; the results were recorded in cm. The test was repeated three times with a 60-s rest period after every trial. After that, the average result was the final score.

### 2.4. Statistical Analysis

The results are expressed as means ± SD and 95% confidence intervals (95% CI). The Kolmogorov–Smirnov test was used to validate the assumption of normality. A one-way repeated measures ANOVA was conducted to investigate the difference between no tape and different Kinesio Taping^®^ applications. The level of statistical significance was set at *p* < 0.05. IBM-SPSS 20.0 (IBM, Inc., Chicago, IL, USA) was used for statistical analysis. 

## 3. Results

A total of sixteen participants completed the present study. No significant differences were found between the interventions, neither in the characteristics information nor in the outcome measures at baseline. Table 1 and Table 2 show no significant differences in jump, flexibility, and running tests (*p* > 0.05). Additionally, no significant differences existed in any outcome measures between the interventions (*p* > 0.05). 

## 4. Discussion

To our knowledge, this is the first study that examines the effects of KT on functional performance in healthy soccer players. The results of this study showed no significant acute effects on functional performance after each Kinesio tape application compared with no tape intervention. Specifically, we did not observe any enhancement in jump, flexibility, and running tests in all conditions analysed (*p* > 0.05). Our results are in line with a previous study which observed no changes in jump performance in healthy female athletes before and after Kinesio tape application [36]. 

Generally, the mechanism of the KT application that can improve athletic performance has not yet been fully explained. A systematic review by Williams et al. (2012) investigated treatment and prevention methods for sports injuries and reported small beneficial influences for improving strength and muscle activity [37,38]. On the other hand, enhanced tactile input of the skin [31] and fascial unloading [11] were suggested as possible mechanisms for improving strength and jump performance. However, an investigation with soccer athletes confirmed our findings. The absence of performance benefits in soccer athletes compared with untrained individuals may be due to the clear recruitment of the skeletal muscle fibres during maximal exercise. Hence, it is probably more difficult for the sensory input of the skin to modulate the performance of muscles [39]. Moreover, the KT application time required to develop a peak effect remains unknown. The evidence showed immediate strength effects on the gastrocnemius muscle, while the utilization of KT application for hamstring muscles increases peak force with a delay of two days [11].

The current study obtained a result similar to Lins et al. (2013), who reported no significant changes in quadriceps flexibility in healthy untrained individuals [40]. Merino-Marban et al. (2011) and Krohn et al. (2011) did not report any significant difference in hamstring flexibility immediately after KT application in healthy individuals [7,41]. Additionally, Taradaj et al. (2015) reported no improvement in the flexibility of quadriceps muscle immediately following facilitative KT application in volleyball athletes [42]. Thus, it is likely that facilitative KT application has no acute effect on muscle flexibility in healthy subjects.

Running performance is determined by various muscular factors such as morphology, muscular size, firing frequency, and unit recruitment [43]. Our findings showed no acute changes in running performance after KT application—specifically, contact time, flight time, stride, and rhythm. Generally, the mechanism by which KT application improves athletic running performance remains unclear. Our findings are contrary to the study by Pelletier et al. (2017), who reported that KT application influences kinematic run-in individuals with patellofemoral pain syndrome (PFPS) [44]. Individuals in the study by Pelletier et al. (2017) were represented with PFPS compared with our trained athletes [44]. Soccer athletes have shown limitations in their ability to benefit from differences in their conditions since their running performance level is close to their limits for further adaptation [45,46]. This may explain the conflict between our results and those obtained by Pelletier et al. (2017), who included PFPS individuals [44]. 

Kase et al. (2003) explained the method of KT application to achieve the desired effects [3]. However, the KT application methods in numerous studies are variable. Many studies [31,32,39,40,41,46] that aimed to enhance strength, flexibility, and jump abilities when applying KT from proximal to distal, as recommended by Kase et al. (2003), failed to achieve the desired effects [3]. A study using a combination of activating and deactivating KT application techniques reported improvements in performance for both techniques [11]. Furthermore, there are discrepancies regarding the degree of KT tension for its application. Again, most researchers refer to the original technique described by Kase et al. (2003), who recommended a maximum stretching of the muscles and a tape application without tension for muscle activation [3]. However, some researchers have applied KT with tension and without clear stretching of the muscles [39], some with tension and stretching [3], and some have provided no details on the subject [32].

The present study only examined the acute effects of KT application on functional performance. So, the chronic effects of KT application on athletic functional performance still need to be clarified. Furthermore, there is no general agreement either about the influence of KT application or the accurate application technique and the time needed to achieve the desired effects. In line with the study by Nunes et al. (2020) [32], we do not support the use of Kinesio taping to improve functional performance in healthy athletes. 

This study has many limitations that should be mentioned. First, only healthy soccer players were included. Thus, we cannot generalize our findings to nonathletic individuals and those in other athletic fields. Second, the design of this study was not double-blind. However, using an instrumented measure for outcomes assessment and blinding the clinical assessor to the participants partially limited the bias.

## 5. Conclusions

Our findings indicate no significant differences in running, flexibility, and jump abilities immediately after facilitative KT application compared with the no-tape condition in healthy athletes. In this context, KT application does not appear to be useful in improving functional performance immediately in healthy athletes, specifically soccer players. However, further studies with increasing application time and using inhibition application of KT are strongly recommended to understand the effect of KT application on athletic performance.

## Figures and Tables

**Table 1:** Data for the jump test, sit and reach test, and running test are expressed as mean ± SD and 95% confidence intervals.

**Jump test CMJ (cm)**	**Mean ± SD**	**95% CI**
No tape	36.38 ± 4.66	33.89 to 38.86
H	35.69 ± 5.00	33.02 to 38.35
Q	35.81 ± 4.55	33.39 to 38.24
H + Q	35.87 ± 4.94	33.24 to 38.50
**Sit and reach flexibility (cm)**		
No tape	2.94 ± 8.27	−1.47 to 7.34
H	3.31 ± 7.73	−0.81 to 7.43
Q	3.69 ± 8.15	−0.65 to 8.02
H + Q	3.60 ± 8.08	−0.71 to 7.89
**Running test contact time (s)**		
No tape (cm)	0.282 ± 0.017	0.273 to 0.291
H (cm)	0.285 ± 0.017	0.277 to 0.294
Q (cm)	0.287 ± 0.018	0.278 to 0.297
H + Q (cm)	0.285 ± 0.017	0.276 to 0.295
**Running test fly time (s)**		
No tape	0.083 ± 0.015	0.075 to 0.091
H	0.080 ± 0.018	0.070 to 0.089
Q	0.080 ± 0.021	0.069 to 0.091
H + Q	0.079 ± 0.017	0.070 to 0.087
**Running test rhythm (step/s)**		
No tape	2.76 ± 0.13	2.68 to 2.82
H	2.74 ± 0.13	2.67 to 2.81
Q	2.73 ± 0.12	2.66 to 2.79
H + Q	2.76 ± 0.11	2.69 to 2.81
**Running test stride (m)**		
No tape (cm)	2.22 ± 0.11	2.16 to 2.27
H (cm)	2.22 ± 0.12	2.15 to 2.28
Q (cm)	2.23 ± 0.11	2.17 to 2.28
H + Q (cm)	2.21 ± 0.09	2.16 to 2.26

H = hamstring muscle; Q = quadriceps muscle; H + Q = hamstring and quadriceps muscles.

**Table 2:** Comparisons between no tape and different Kinesio tape^®^ applications with respect to average jump test, sit and reach test, and running test data. Data are presented as mean difference and 95% confidence interval of the difference (*p* = statistical difference between means).

**Jump test CMJ (cm)**	**Mean Difference**	**95% CI**	***p***
No tape vs. H	0.69	−0.77 to 2.15	1.00
No tape vs. Q	0.56	−0.35 to 1.48	0.49
No tape vs. H + Q	0.50	−0.77 to 1.77	1.00
**Sit and reach flexibility (cm)**			
No tape vs. H	−0.37	−1.96 to 1.21	1.00
No tape vs. Q	−0.75	−2.40 to 0.90	1.00
No tape vs. H + Q	−0.66	−2.58 to 1.27	1.00
**Running test contact time (s)**			
No tape vs. H	−0.004	−0.010 to 0.003	0.98
No tape vs. Q	−0.005	−0.011 to 0.001	0.14
No tape vs. H + Q	−0.004	−0.010 to 0.003	0.99
**Running test fly time (s)**			
No tape vs. H	0.004	−0.006 to 0.013	1.00
No tape vs. Q	0.003	−0.006 to 0.012	1.00
No tape vs. H + Q	0.005	−0.004 to 0.014	0.98
**Running test rhythm (step/s)**			
No tape vs. H	0.013	−0.028 to 0.055	1.00
No tape vs. Q	0.025	−0.030 to 0.081	1.00
No tape vs. H + Q	−0.001	−0.042 to 0.040	1.00
**Running test stride (m)**			
No tape vs. H	0.001	−0.045 to 0.046	1.00
No tape vs. Q	−0.009	−0.064 to 0.047	1.00
No tape vs. H + Q	0.002	−0.029 to 0.033	1.00

H= hamstring muscle; Q = quadriceps muscle; H + Q = hamstring and quadriceps muscles.

## Data Availability

Not applicable.
